# Effects of Newcastle Disease Virus Strains AF2240 and V4-UPM on Cytolysis and Apoptosis of Leukemia Cell Lines

**DOI:** 10.3390/ijms12128645

**Published:** 2011-11-30

**Authors:** Aied M. Alabsi, Siti Aishah Abu Bakar, Rola Ali, Abdul Rahman Omar, Mohd Hair Bejo, Aini Ideris, Abdul Manaf Ali

**Affiliations:** 1 Department of Biotechnology, Faculty of Agriculture and Biotechnology, University Sultan Zainal Abidin (UniSZA), Terengganu 20400, Malaysia; E-Mails: sitiaishah@unisza.edu.my (S.A.A.B.); rola_absi@yahoo.com (R.A.); 2 Department of Cell and Molecular Biology, Faculty of Biotechnology and Biomolecular Sciences, University Putra Malaysia (UPM), Serdang 43300, Malaysia; 3 Faculty of Dentistry, University of Malaya (UM), Kuala Lumpur, 50603, Malaysia; 4 Faculty of Veterinary Medicine, University Putra Malaysia (UPM), Serdang 43300, Malaysia; E-Mails: aro@vet.upm.edu.my (A.R.O.); mdhair@vet.upm.edu.my (M.H.B.); aiini@vet.upm.edu.my (A.I.); 5 Institute of Bioscience, University Putra Malaysia (UPM), Serdang 43300, Malaysia

**Keywords:** NDV, cytolytic, apoptosis, flow-cytometry, leukemia

## Abstract

Newcastle disease virus (NDV) is used as an antineoplastic agent in clinical tumor therapy. It has prompted much interest as an anticancer agent because it can replicate up to 10,000 times better in human cancer cells than in most normal cells. This study was carried out to determine the oncolytic potential of NDV strain AF2240 and V4-UPM on WEHI-3B leukemia cell line. Results from MTT cytotoxicity assay showed that the CD_50_ values for both strains were 2 and 8 HAU for AF2240 and V4-UPM, respectively. In addition, bromodeoxyuridine (BrdU) and trypan blue dye exclusion assays showed inhibition in cell proliferation after different periods. Increase in the cellular level of caspase-3 and detection of DNA laddering using agarose gel electrophoresis on treated cells with NDV confirmed that the mode of cell death was apoptosis. In addition, flow-cytometry analysis of cellular DNA content showed that the virus caused an increase in the sub-G1 region (apoptosis peaks). In conclusion, NDV strains AF2240 and V4-UPM caused cytolytic effects against WEHI-3B leukemic cell line.

## 1. Introduction

Leukemia is a malignant neoplasm, characterized by neoplastic proliferation of hematopoietic cells, namely their diffuse replacement of the normal bone marrow [1,2]. Chemotherapeutic agents are used to prevent leukemic cells from multiplying, invading, metastasizing and finally killing the patient but they are very toxic and inhibit replication of both cancer and normal cells [3]. Over the past century, a series of case studies and anecdotal reports have indicated that viruses could indeed be used as anticancer therapeutics [4]. NDV is one of the nonengineered oncolytic viruses, which has a long history as a broad-spectrum oncolytic agent that can destroy tumor cells and stimulate the immune system [5]. NDV possesses several unique properties which make it an excellent anticancer agent; it has good cell binding properties, it binds specifically to tumor cells, it replicates selectively in tumor cell cytoplasm, it is relatively safe and it can act as an adjuvant [6]. It has oncolytic activity that can destroy tumor cells and stimulate the immune system. Many strains of NDV (73-T, MH68, Italian, Ulester, Rokin, PV701 and HUJ) have been shown to exhibit oncolytic activity [5,7]. In addition, the oncolytic effects of six Malaysian strains of NDV (AF2240, 01/C, Ijuk, S, F, and V4) have also been studied on several tumor cell lines [5,7]. However, no studies have yet been made using NDV oncolytic activity on myelomoncytic leukemia. Therefore, in this study the oncolytic effects of two Malaysian local NDV strains AF2240 and V4-UPM were tested *in vitro* against Mouse myelomoncytic leukemia cell line, WEHI-3B.

## 2. Results

### 2.1. Cytotolytic Effects of NDV on Normal Cells and Myelomonocytic Leukemia Cells

In this study, the cytolytic effects of Newcastle disease virus strains AF2240 and V4-UPM on mouse myelomonocytic leukemia (WEHI-3B), promyelocytic leukemia (HL-60) and T-lymphoblastoid leukemic (CEM-SS), normal mouse fibroblast (3T3), mouse spleen lymphocyte and A peripheral blood mononuclear cell (PBMC) were determined by measuring the cytotoxic dose that kill 50% of the cell population as compared to the untreated control for various periods using colorimetric cytotoxicity assay (MTT). The assay for each strain was repeated three times. Both AF2240 and V4-UPM strains showed cytolytic effect on WEHI-3B cell lines in dose-dependent manner. The titer of virus that killed 50% (CD_50_) of WEHI-3B cells, compared to untreated cells after 72 h of treatment were 2 ± 0.2 HAU and 8 ± 0.2 HAU (Haemagglutinating Units) for the AF2240 strain and V4-UPM strain, respectively. Furthermore, both NDV strains showed cytolytic effect on HL-60 and CEM-SS human leukemia cell lines (Table 1). On the other hand, AF2240 and V4-UPM strains showed low cytotoxic effect (CD_50_) on normal mouse fibroblast (3T3), mouse spleen lymphocyte and peripheral blood mononuclear cell (PBMC) cells, which were used as normal cells (Table 1).

### 2.2. BrdU Cell Proliferation Assay

**Figure 1:**
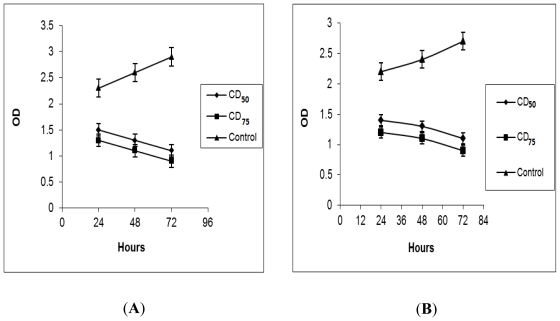
BrdU Proliferation assay, effects of different concentrations of Newcastle disease virus (NDV) strains AF2240 (**A**) and V4-UPM (**B**) on the proliferation and viability of WEHI-3B cells.

The effects of NDV strains AF2240 and V4-UPM on cell proliferation of WEHI-3B cells based on the DNA synthesis phase were investigated using BrdU cell proliferation assay. This assay showed decrease in 570 nm optical density (OD) of WEHI-3B cells after treated with NDV strains AF2240 and V4-UPM in a time and concentration-dependent manner (Figure 1). As observed in Figure 1, untreated WEHI-3B cells exhibited an increase in OD from day 1 to 3. However, the WEHI-3B cells treated with CD_50_ and CD_75_, showed decrease in OD from day 1 to 3.

Similarly, as shown in the Figure 1, OD values in treated cells with CD_75_ decreased more than CD_50_ treatment until the third day. The decreased OD post treatment at both concentrations from first to third day was statistically significant (*p* < 0.05) when compared against the negative control.

### 2.3. Trypan Blue Exclusion Assay

**Figure 2:**
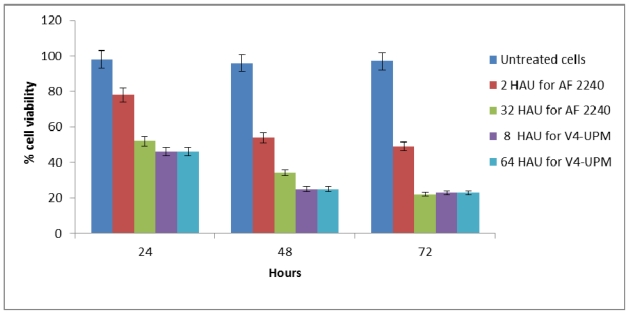
Trypan blue exclusion assay. The percentage of viable cells in WEHI-3B cell population after treatment with different concentrations of virus strains at various time intervals.

Based on the results of the trypan blue dye exclusion assay, increasing NDV exposure time had a significant effect on WEHI-3B cell viability. Treatment of WEHI-3B cells with CD_50_ and CD_75_ concentrations of virus strains (2 and 32 HAU for AF2240 and 8 and 64 HAU for V4-UPM) resulted in a dose- and time-dependent inhibition of cell viability. As observed in Figure 2, at high concentration (CD_75_) of AF2240, WEHI-3B cell viability reduced to 52% at 24 h as compared to the control and finally declined to 34% and 22% after 48 and 72 h, respectively. At CD_50_ concentration of AF2240, WEHI-3B cell viability reduced to 78% at 24 h as compared to the control and finally reduced to 54% and 49% after 48 and 72 h, respectively. On the other hand, at high concentration CD_75_ of V4-UPM, WEHI-3B cell viability reduced to 46% at 24 h as compared to the control and finally declined to 25% and 23% after 48 and 72 h, respectively. Whereas, at CD_50_ concentration of V4-UPM, WEHI-3B cell viability reduced to 66% at 24 h compared to the control and finally declined to 56% and 50% after 48 and 72 h, respectively.

### 2.4. DNA Fragmentation Assay

**Figure 3:**
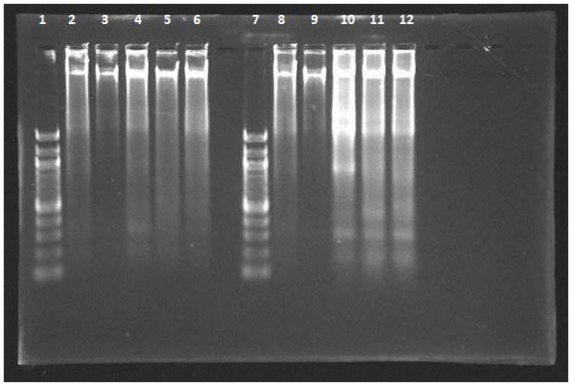
Agarose-gel-electrophoretic patterns showing DNA fragmentation of WEHI-3B cells treated with NDV strains AF2240 and V4-UPM at CD_50_ concentrations. From left to right: lane **1**,**7**: 100 bp DNA markers; lane **2**,**8**: WEHI-3B cell treated with doxorubicin (8 μg/mL); lane **3**,**9**: untreated WEHI 3B cell; lane **4**–**6**: WEHI-3B cell treated with AF2240 strain after 24, 48, and 72 h, respectively; lane **10**–**12**: WEHI-3B cell treated with V4-UPM strain after 24, 48, and 72 h, respectively.

Fragmentation of chromosomal DNA is the biological hallmark of apoptosis. During apoptotic cell death, cellular endonucleases cleave genomic DNA between nucleosides producing fragments whose lengths vary by multiples of 180–200 bp. When resolved using agarose gel electrophoresis, these DNA fragments appear as a nucleosomal ladder In this study, both NDV strains AF2240 and V4-UPM caused DNA fragmentation in WEHI-3B cells at CD_50_ values, 2 and 8 HAU for AF2240 and V4-UPM, after 24, 48 and 72 h. DNA fragmentation of 180–200 base pairs multiples appeared as distinctive ladder-like pattern on an agarose gel (Figure 3).

### 2.5. Effects of NDV on Cell Cycle Distribution

**Figure 4:**
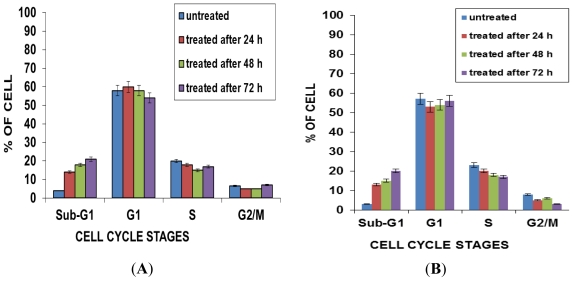
Flow-cytometry Cell cycle analysis of WEHI-3B cell population after staining with propidium iodide. (**A**) Cells treated with NDV strain AF2240 at CD_50_ value; (**B**) cells treated with NDV strain V4-UPM at CD_50_ value. Note: Each value represents of the mean of three replication ± SEM, (*p* < 0.05).

**Figure 5:**
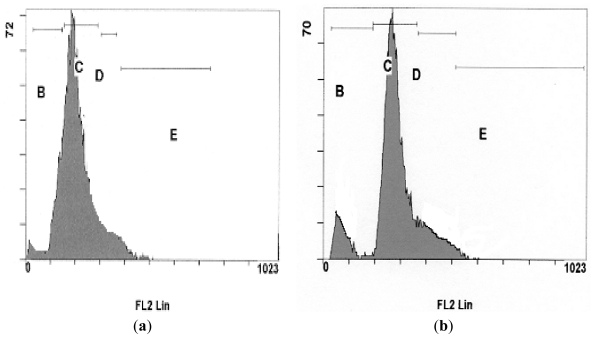

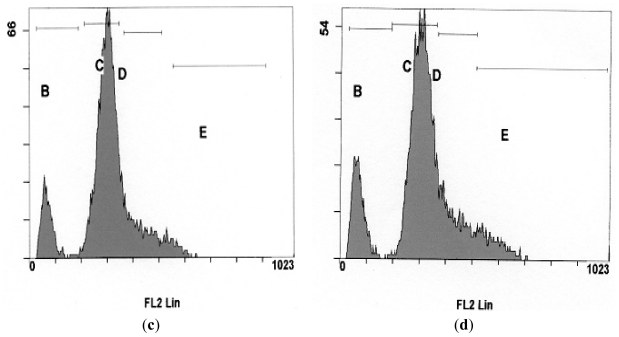
DNA fluorescence histograms of WEHI-3B cells treated with NDV strain AF2240 at CD_50_ value (**a**) untreated cells; (**b**) cells treated after 24 h; (**c**) cells treated after 48 h; (**d**) cells treated after 72 h. (**B**) Sub-G1; (**C**) G1; (**D**) S; and (**E**) G2/M.

**Figure 6:**
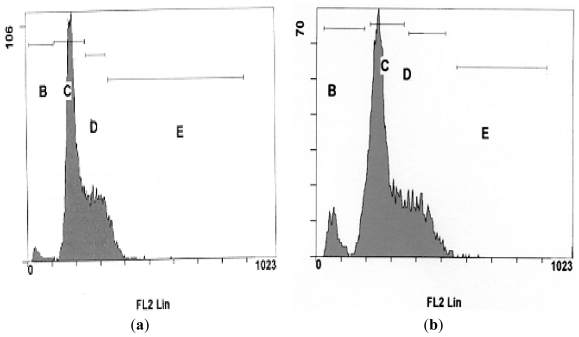

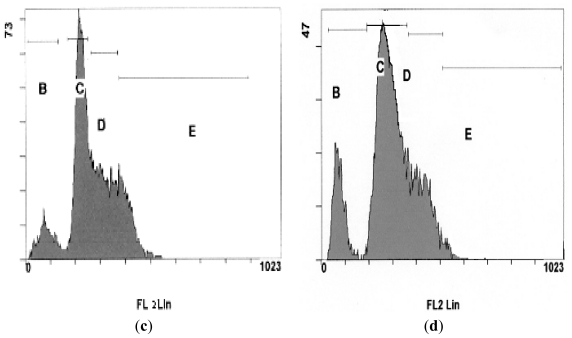
DNA fluorescence histograms of WEHI-3B cells treated with NDV strain V4-UPM at CD_50_ value (**a**) untreated cells; (**b**) cells treated after 24 h; (**c**) cells treated after 48 h; (**d**) cells treated after 72 h. (**B**) Sub-G1; (**C**) G1; (**D**) S; and (**E**) G2/M.

In this study, quantification of apoptosis on the basis of nuclear changes was performed by staining apoptotic nuclei with propidium iodide (PI). FACS analysis was used to study the cell cycle kinetics of NDV strains AF2240 and V4-UPM (CD_50_) on WEHI-3B cells, whereby 20,000 cells were analyzed using the Cell Quest Software. As shown in Figures 4, 5 and 6, after 24 h treatment with CD_50_ values of NDV strains AF2240 and V4-UPM, both virus strains were able to induce 14% and 13% of apoptotic cells of WEHI-3B cells population and the number of apoptotic cells started to increase gradually with time. After 48 and 72 h of treatment, NDV strain AF2240 was able to induce 18% and 21% of apoptotic cells (Figure 4A) while NDV strain V4-UPM able to induce 15% and 20% after 48 and 72 h (Figure 4B). There was a significant increase in apoptosis (Sub-G1) induction with time (*p* < 0.05) of WEHI-3B cell treated with both NDV strains compared to untreated cells. On the other hand both NDV strains showed no arrests of WEHI-3B cells at specific cell cycle phase, meaning NDV was able to kill WEHI-3B cells at different cell cycle stages. This virus-induced cell cycle perturbation concurred with the previous results, suggesting that WEHI-3B cells undergo apoptosis more extensively with increasing time.

### 2.6. Activation of Caspase-8, an Initiator Caspase for the Extrinsic Pathway

**Figure 7:**
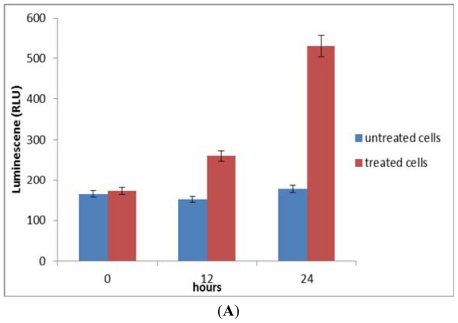

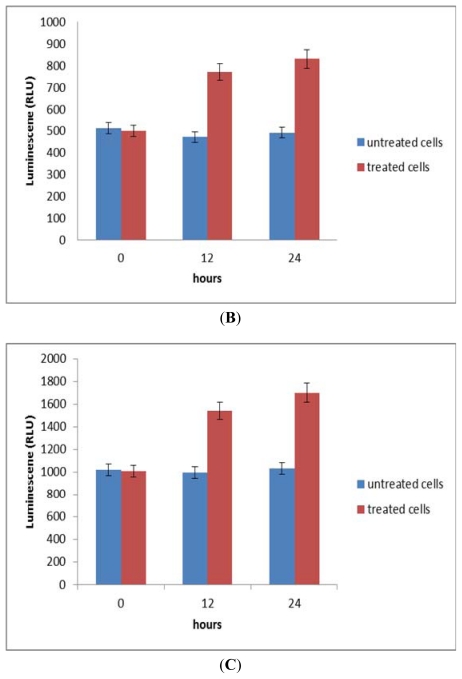
Effects of NDV on caspase 8 (**A**), caspase 9 (**B**) and caspase 3/7 (**C**) in WEHI-3B cells. Data were expressed as mean ± SEM of three experiments (*n* = 3). Significant differences from untreated control are indicated by *p* < 0.05.

The involvement of caspase-8 in response to the treatment of NDV was determined. Treatment for 12 h activated caspase-8 at 26.8% and increased to 59.1% at 24 h post-treatment (Figure 7A).

### 2.7. Activation of Caspase-9, an Initiator Caspase for the Intrinsic Pathway

The involvement of caspase-9 in response to the treatment of NDV was also examined. As shown in Figure 7B, there was a gradual increase (from 12 to 24 h) of caspase-9 activation at 46.5 and 55.8%, respectively.

### 2.8. Activation of Effector/Executioner Caspases (Caspase-3/7)

The involvement of effector caspase, caspase-3/7 in response to the treatment of NDV was further determined. Treatment of WEHI-3B cells with NDV for 12 h activated caspase-3/7 at 42.67% and increased to 78.4% at 24h post-treatment (Figure 7C).

## 3. Materials and Methods

### 3.1. Propagation and Purification of NDV Strains AF2240 and V4-UPM

NDV was propagated in allantoic fluid of 9–11 days-old embryonated chicken eggs at 37 °C for 48 h. The allantoic fluid was harvested and the presence of virus was confirmed by the haemaglutination test [8]. NDV purified as previously described by Chambers and Samson (1980) [9].

### 3.2. Cells and Cell Culture

(WEHI-3B) murine myelomoncytic leukemia cell line and 3T3 (Mouse fibroblast) cell lines were provided by the Laboratory of Molecular and Cell Biology, Institute of Bioscience, Universiti Putra Malaysia. WEHI-3B and 3T3 cell lines were cultured in DMEM (Sigma, USA) containing 10% fetal bovine serum (FBS), 100 U/mL penicillin and 100 μg/mL streptomycin at 37 °C in a humidified atmosphere of 5% CO_2_ in air. The cells were grown to confluence and sub-cultured at three to four days interval before the experiments.

### 3.3. Cell Viability and Proliferation Assessment

#### 3.3.1. MTT Cytotoxicity Assay

Leukemia cells inhibition by NDV was measured using Microtitration cytotoxicity [10,11]. About 150 μL complete medium were added into of flat-bottom 96–well plate (Nunclon™, Denmark) and 50 μL of the 2-folded serial virus dilution were added into the wells. In the last well, 50 μL of PBS were added instead of the virus, which represented as control. Then 50 μL of 5 × 10^5^ cells of CEM-SS (human T-lymphobalstic leukemia), WEHI-3B (Mouse Myelomoncytic leukemia), HL-60 (Promyelocytic leukemia), were added to top up the final volume to 200 μL and the plate was incubated at 37 °C in an atmosphere of 5% CO_2_. Seventy-two hours later, 20 μL of MTT (5 mg/mL) in PBS solution was added to each well then the plate was further incubated for 4 h. Most of the medium was removed and 100 μL of DMSO (dimethyl sulfoxide) was added into the wells to soluble the crystals. Finally the OD was measured by (ELISA) reader at wavelength of 570 nm. Then graphs of percentage of viable cells *versus* virus titer HAU were plotted. The value of CD_50_ was determined from the graphs obtained at the concentration that cause 50% cell reduction as compared with controls.

#### 3.3.2. BrdU Proliferation Assay

The BrdU proliferation assay for treated and untreated WEHI-3B cells was carried out using a kit BrdU Cell Proliferation assay (CHEMICON, USA). WEHI-3B cells at concentration of 1 × 10^5^ cells/mL treated with both NDV strains at CD_50_ and CD_75_ concentrations (2 and 32 HAU for AF2240 and 8 and 64 HAU for V4-UPM). Then the plates were incubated in an atmosphere of 5% CO_2_ at 37 °C for 24, 48 and 72 h. After incubation periods, the cells were washed with PBS twice. Further procedure was done according to protocol specified by the manufacturer. The plates were read using a spectrophotometer microplate reader at dual wavelength of 450 nm and 550 nm. The OD of samples was plotted against time to determine the growth rates of cells in a given value.

#### 3.3.3. Trypan Blue Exclusion Assay

Trypan Blue Exclusion assay was employed to determine the number of viable cells in cultures. The cells were incubated with different CD_50_ and CD_75_ concentrations of both NDV strains (2 and 32 HAU for AF2240 and 8 and 64 HAU for V4-UPM) at 37 °C. The viability of the cells was then determined at the designated time interval. Cells were analyzed by viable cell counts and the percentage of cell viability was obtained. The results were expressed as the mean percentage of cell viability ± SEM of triplicate cultures.

### 3.4. DNA Fragmentation Assay

Cells at a concentration of 5 × 10^6^ cells/mL were seeded into 6-well plate (Nunclon^TM^, Denmark) in 2 mL culture medium with a concentration of CD_50_ value of viruses. Some wells were left with no virus to be used as a control. After the 72 h of incubation, cells were spun down at 1000 rpm for 10 min and the pellet was washed with PBS twice. The DNA extraction from treated and untreated cells was carried out according to protocol of a kit for Blood and Cultured Cells from QIAGEN. The purified DNA was subjected to electrophoresis in 1.5% agarose gel. The gel was electrophoresed for and stained in ethidium bromide. The bands were visualized using UV light transillumintor.

### 3.5. Analysis of Cellular DNA Content Using Propidium Iodide

Cells at a concentration of 5 × 10^6^ cells/mL of WEHI-3B cell line was incubated in 6-well plate in with a concentration of CD_50_ value of virus for 72 h. Some wells were left with no virus to be used as a control. After the incubation period, the Cells were fixed by adding 500 μL of 80% cold ethanol and kept for at least 2 h at −20 °C. Cells were pelleted at 1000 rpm for 10 min and the ethanol was discarded. The cell pellet was washed with 1 mL (PBS/sodium azide) twice. The pellet was resuspended with 1 mL of (PBS + 0.1% triton X-100 + 10 mm EDTA + 50 μg/mL RNase + 2 μg/mL Propidium iodide) followed by incubated for 1/2 to 1 h at 4° C. Finally, samples were analyzed by flow cytometer (Beckman Coulter, USA).

### 3.6. Analysis of Caspase-3/7, 8 and 9 Activities

Caspase-3/7, 8 and 9 activities was measured using Caspase-Glo^®^ 3/7, 8 and 9 Assay Kit (Promega, USA). The kit provided a lumigenic caspase-3/7, 8 and 9 substrates, in a reagent optimized for caspase activity, luciferase activity and cell lysis. Cells of WEHI-3B were grown in white-wall, optical bottom 96-well plate and treated with NDV at CD_50_ for 0, 12 and 24 h. After the incubation time, an equal volume of the reagent were added to the cells and further incubated for 1 h. The contents were mixed gently using a plate shaker at 300 to 500 rpm for 30 s. Luminescene plate reader, Tecan (Infinite M200) was used to measure luminescene intensity. Blank values were subtracted from experimental values.

### 3.7. Statistical Analysis

Data was expressed as mean ± SEM. Statistical analysis was performed with Student’s *t*-test for data from MTT cytotoxicity assay, BrdU Proliferation Assay, Trypan blue exclusion assay, flowcytometry and caspases with p value of less than 0.05.

## 4. Discussions

Oncolytic viruses are viruses that infect and replicate in cancer cells, destroying these harmful cells and leaving normal cells largely unaffected. NDV is an oncolytic virus with the ability to induce tumor lysis through different mechanisms [12] and it appears to replicate and kill tumor cells but not normal human cells [13]. Development of biologically targeted agents that exploit differences between cancerous and normal cells and permit greater specificity for cancer cells with less damage to normal cells is still the ultimate goal in the field of antineoplastic drug discovery [14]. This study showed that NDV AF2240 and V4-UPM strains have the ability to induce cytolysis, apoptosis and anti-proliferation in (WEHI-3B) murine myelomoncytic leukemia cell line.

Based on the MTT assay, treatment with both NDV strains resulted in concentration- and time-dependent cytolyitic effects on the WEHI-3B cells but not on mouse fibroblast (3T3), mouse spleen lymphocyte and peripheral blood lymphocyte cells, which were used as normal cells. These results complies with previous other studies [7,15–18] which stated that the effectiveness of NDV strains AF2240 and V4 UPM as an oncolytic agent was found on breast cancer cell lines (MCF-7 and MDA-231), leukemia cell lines (HL-60 and CEM-SS) and brain tumor cell line (DBTRG.5MG and U87MG).

To further investigate the cytolytic effects of the virus, inhibition of WEHI-3B proliferation was determined using the bromodeoxyuridine (BrdU) cell proliferation assay, which is based on the DNA synthesis phase [19]. Inoculation of both NDV strains on the WEHI-3B cells with high titre showed that the growth rates of the cells decreased more than those inoculated with low titre. On the other hand, untreated WEHI-3B cells exhibited an increase in the growth rates. Therefore, both NDV strains were able to inhibit WEHI-3B cell proliferation.

The trypan-blue exclusion test also showed that NDV has significant antiproliferative effects on WEHI-3B cells. This test is based on the principle that living cells possess intact cell membranes that exelude the dye, whereas dead cells do not [20].

Apoptosis is particularly relevant to viral pathogenesis; it is a major mechanism for viral clearance by the mammalian immune system which induces apoptosis in infected cells. It is becoming increasingly apparent that many viruses have evolved proteins that are capable of attenuating apoptosis [21]. A number of methods have been used by researchers to distinguish between apoptosis and necrosis. In this study we reported that both NDV strains stimulate DNA fragmentation characteristic of apoptosis in WEHI-3B cell line at 24, 48 and 72 h post-inoculation. Internucleosomal cleavage of DNA is likely to be in the later phase of apoptotic process [22–24]. The DNA ladder assay is generally accepted as specific for apoptosis because it detects oligonucleosomal cleavage rather than artificial DNA cleavage or necrosis. To date, apoptosis has been characterized biochemically by the production of 180–200 bp internucleosomal DNA fragments resulting from the activation of an endonuclease [23].

Further confirmation of the mode of cell death was carried out by Flow cytometric analysis of cell cycle which is a rapid and quantitative measure on apoptotic changes cells by staining with DNA dyes [25]. This method is useful for quantitative estimates of the fractions of cells in the different phases of the cell cycle [26]. Untreated and treated WEHI-3B cell lines were evaluated for apoptosis by measuring the amount of apoptotic cells using of DNA flow cytometry (FCM). In the present study, we found that an apoptotic peak appeared before the G1 phase when WEHI-3B cells were treated with both NDV strains. Both NDV strains caused an increase in the subG_1_ region which increased of time. This time-dependent effect was significantly apparent upon the induction of apoptosis, with no induction of cell cycle arrest in any specific phase. The fluorescence histograms showed that there were less of treated WEHI-3B cells in all three phases of the cell cycle (G_1_, S and G_2_/M) accompanied by a large increase in the sub-G1 region (apoptosis peak).

Caspases are effectors and executioners. The effector caspases can be activated by multiple pathways, some involving the mitochondria and others independent of this organelle. Once effector caspases become active, they cleave and activate the downstream executioner caspases. These downstream caspases then cleave and activate a series of molecules, which are involved in terminal apoptotic events. Without proper functioning caspases, the upstream tumor-suppressor genes may be unable to activate the apoptotic pathway. Therefore, caspases are an important indicator for tumor therapy. In this study, Caspase-8 was identified as an initiator caspase triggered by death receptors. So, the activation of caspase-8 suggested that NDV might induce apoptosis through the extrinsic death receptor-pathway. Death receptors are cell-surface cytokine receptors belonging to the tumor necrosis factor (TNF) receptor superfamily that trigger apoptosis after binding to a group of structurally related ligands or specific antibodies [27–29]. The activation of caspase-9 in NDV-treated WEHI-3B cells can be related with the involvement of mitochondria. Mitochondria act as important sensors of cellular damage. Any increase in the permeability of the outer mitochondrial membrane can allow certain proteins, such as cytochrome *c*, to be released from the mitochondrial intermembrane space into the cytosol and activate the apoptosome. Upon activation within the apoptosome, caspase-9 then propagates the caspase cascade through activation of caspases-3 and -7. Caspase-3 in turn activates caspases-2 and -6 and then promotes activation of the downstream caspases-8 and -10 [30]. Caspase-3 also participates in a feedback amplification loop to further process caspase-9 [31]. Thus, it is evident that once caspase-9 is activated within the apoptosome, there is a rapid amplification of the death signal through the activation of a panoply of other caspases. The executioner caspase include caspases-3, -6 and -7. Many studies have suggested that caspase-3 is the primary executioner caspase with relatively few physiological substrates for caspases-6 and -7 [32]. It is assumed that caspase-3 has a position as a central executioner caspase in mammals and the role of caspase-3 is supported by the convergence of both death-receptor and mitochondrial-mediated death pathways at caspase-3 activation as well as by the wide range of potential caspase-3 substrates [33,34].

As a conclusion, we investigated the use of NDV strains AF2240 and V4-UPM as an oncolytic agent of WEHI-3B myelomoncytic leukemia cells. Our study suggests that the NDV have a potent effect on leukemia cells and that its cytotoxicity increases with increasing titers of the virus. NDV was also found to act by inducing apoptosis in the WEHI-3B leukemia cells.

## Figures and Tables

**Table 1:** Cytotoxic effects dose (CD_50_) of Newcastle disease virus strains (AF2240 and V4-UPM), in comparison with commercial drugs (doxorubicin and arabinocytocine) against leukemia and normal cell lines at 72 h of incubation.

Cell Lines	AF 2240	V4-UPM	Doxorubicin	Arabinocytocine (Ara-C)
WEHI-3B	2 ± 0.2	8 ± 0.2	0.8 ± 0.6	1.0 ± 0.13
HL-60	25 ± 0.3	110 ± 0.8	1.8 ± 0.1	0.3 ± 0.05
CEM-SS	16 ± 0.6	64 ± 0.5	3.5 ± 0.1	3.8 ± 0.30
3T3	NO CD_50_	NO CD_50_	7.0 ± 0.4	2.8 ± 0.20
PBMC	NO CD_50_	NO CD_50_	11.0 ± 0.8	7.0 ± 0.80
Mouse spleen lymphocyte	NO CD_50_	NO CD_50_	6.5 ± 0.2	4.7 ± 0.6
